# Targeting the DNA Repair Endonuclease ERCC1-XPF with Green Tea Polyphenol Epigallocatechin-3-Gallate (EGCG) and Its Prodrug to Enhance Cisplatin Efficacy in Human Cancer Cells

**DOI:** 10.3390/nu10111644

**Published:** 2018-11-03

**Authors:** Joshua R. Heyza, Sanjeevani Arora, Hao Zhang, Kayla L. Conner, Wen Lei, Ashley M. Floyd, Rahul R. Deshmukh, Jeffrey Sarver, Christopher J. Trabbic, Paul Erhardt, Tak-Hang Chan, Q. Ping Dou, Steve M. Patrick

**Affiliations:** 1 Department of Oncology, Karmanos Cancer Institute, Wayne State University, Detroit, MI 48201, USA; jrheyza@med.wayne.edu (J.R.H.); hzhang1@hfhs.org (H.Z.); connerk@karmanos.org (K.L.C.); leiwen314@gmail.com (W.L.); floyda@karmanos.org (A.M.F.); 2 Department of Cancer Biology, University of Toledo Health Science Campus, Toledo, OH 43614, USA; sanjeevani.arora@fccc.edu; 3 Department of Pathology, Wayne State University School of Medicine, Detroit, MI 48201, USA; rdeshmukh@lecom.edu; 4 Department of Pharmacology and Experimental Therapeutics, University of Toledo College of Pharmacy and Pharmaceutical Sciences, Toledo, OH 43614, USA; jeffrey.sarver@utoledo.edu; 5 Center for Drug Design and Development, Department of Medicinal and Biological Chemistry, University of Toledo College of Pharmacy and Pharmaceutical Sciences, Toledo, OH 43614, USA; christrabbic51@gmail.com (C.J.T.); paul.erhardt@utoledo.edu (P.E.); 6 State Key Laboratory of Chemical Biology and Drug Discovery, Department of Applied Biology and Chemical Technology, The Hong Kong Polytechnic University, Hong Kong, China; tak-hang.chan@mcgill.ca; 7 Department of Chemistry, McGill University, Montreal, QC H3A 0B8, Canada; 8 Departments of Oncology, Pharmacology, and Pathology, Karmanos Cancer Institute, Wayne State University, Detroit, MI 48201, USA; doup@karmanos.org

**Keywords:** ERCC1/XPF, cisplatin, DNA repair, chemoresistance, green tea polyphenols

## Abstract

The 5′-3′ structure-specific endonuclease ERCC1/XPF (Excision Repair Cross-Complementation Group 1/Xeroderma Pigmentosum group F) plays critical roles in the repair of cisplatin-induced DNA damage. As such, it has been identified as a potential pharmacological target for enhancing clinical response to platinum-based chemotherapy. The goal of this study was to follow up on our previous identification of the compound NSC143099 as a potent inhibitor of ERCC1/XPF activity by performing an in silico screen to identify structural analogues that could inhibit ERCC1/XPF activity in vitro and in vivo. Using a fluorescence-based DNA-endonuclease incision assay, we identified the green tea polyphenol (-)-epigallocatechin-3-gallate (EGCG) as a potent inhibitor of ERCC1/XPF activity with an IC_50_ (half maximal inhibitory concentration) in the nanomolar range in biochemical assays. Using DNA repair assays and clonogenic survival assays, we show that EGCG can inhibit DNA repair and enhance cisplatin sensitivity in human cancer cells. Finally, we show that a prodrug of EGCG, Pro-EGCG (EGCG octaacetate), can enhance response to platinum-based chemotherapy in vivo. Together these data support a novel target of EGCG in cancer cells, namely ERCC1/XPF. Our studies also corroborate previous observations that EGCG enhances sensitivity to cisplatin in multiple cancer types. Thus, EGCG or its prodrug makes an ideal candidate for further pharmacological development with the goal of enhancing cisplatin response in human tumors.

## 1. Introduction

The use of agents that induce interstrand crosslinks (ICLs), notably the platinum-based analogues, remains a mainstay of cancer treatment and they are used to treat a variety of cancer types including lung, ovarian, and head and neck cancers. Platinum-based drugs induce DNA damage by forming a variety of DNA lesions including ICLs, intrastrand crosslinks, and monoadducts. While platinum-based chemotherapy is often initially effective, resistance to these drugs usually occurs. Resistance to platinums has been attributed to several mechanisms including decreased drug accumulation, loss of mismatch and base excision repair, increased translesion synthesis as well as increased DNA repair [1]. Extensive work devoted to understanding mechanisms of resistance to these therapies has led to the identification of novel drug targets for enhancing therapeutic response and overcoming chemoresistance, including targeting the 5′-3′ structure-specific endonuclease ERCC1/XPF (Excision Repair Cross-Complementation Group 1/Xeroderma Pigmentosum Group F) [2,3]. ERCC1/XPF is a critical complex involved in the repair of DNA ICLs with essential functions in both replication-independent and -dependent ICL repair pathways [4,5]. As such, it is well-established that ERCC1 expression is altered in several tumor types, including lung, head and neck, and ovarian cancers, suggesting its potential use as a drug target or biomarker for response to platinum-based chemotherapy. Indeed, this possibility has been thoroughly investigated in preclinical and clinical studies. Clinical data have shown that low ERCC1 expression is associated with increased overall survival in response to chemotherapy [6]. Further data showed that low ERCC1 expression was associated with a positive response to platinum-based chemotherapy in non-small cell lung cancer patients [7]. Some mixed results have now hampered further clinical development of ERCC1 as a first-in-class platinum biomarker, most notably in a prospective international, randomized Phase III clinical trial where there was an absence of clinical benefit for patients with low ERCC1 receiving a platinum agent [8]. However, these mixed results have been largely attributed to problems pertaining to accurate detection of functional ERCC1/XPF (e.g., antibody specificity and splice variant expression), rather than its usefulness as a potential biomarker for response to platinum-based chemotherapy [9]. Clear evidence from extensive data from biochemical, in vitro, and in vivo approaches suggests critical roles for the complex in repair of platinum-induced DNA damage, suggesting the inhibition of ERCC1/XPF as a potential means of sensitizing tumors to platinum-based chemotherapy.

ERCC1/XPF plays key roles in nucleotide excision repair, ICL repair, and homologous recombination. In the response to DNA-ICLs, ERCC1/XPF plays a key role in incising 5′ to the DNA lesion resulting in the unhooking of the interstrand crosslink from the DNA helix [5]. In both replication-independent and -dependent pathways, ERCC1/XPF-mediated incision appears to be a generally required step for at least a subset of ICLs. In replication-dependent repair of interstrand crosslinks, however, several groups have also postulated that ERCC1/XPF nuclease activity is also essential at a second step downstream from initial unhooking likely during homologous recombination [10]. In line with critical roles for ERCC1/XPF in ICL repair, we have previously shown that siRNA knockdown of ERCC1/XPF could enhance sensitivity of lung cancer cells to cisplatin [3]. Additionally, this sensitivity was induced as a result of decreased DNA repair of interstrand and intrastrand crosslinks.

**Figure 1:**
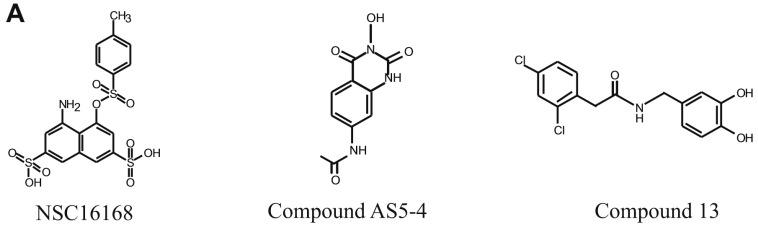

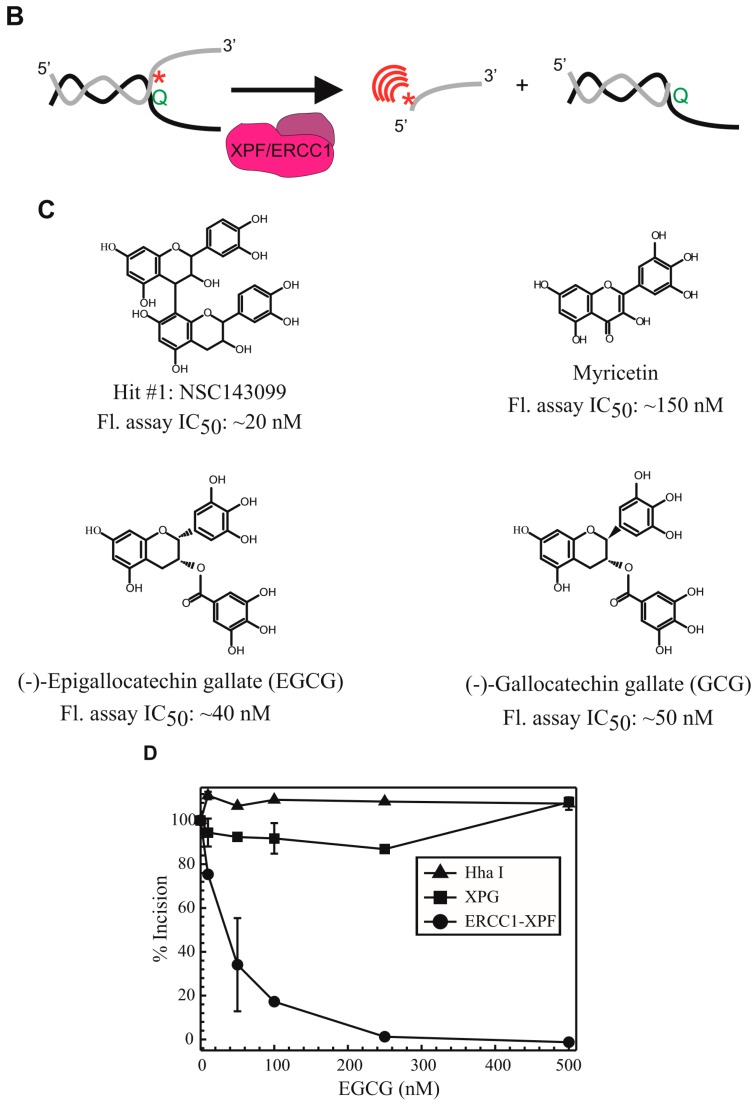
(**A**) Structures of ERCC1/XPF (Excision Repair Cross-Complementation Group 1/Xeroderma Pigmentosum Group F) inhibitors identified in previous studies. The structure of NSC16168 is undergoing further investigation. (**B**) Model of the DNA substrate and product produced by ERCC1/XPF cleavage in the fluorescent–DNA-incision assay. * represents fluorescein and Q represents DABCYL quencher. (**C**) Structure of NSC143099 and the three identified hits from the in silico screen along with their IC_50_s in the DNA-incision assay. (**D**) Plotted results of EGCG-mediated inhibition of ERCC1/XPF in the DNA-incision assay. Results show selectivity for ERCC1/XPF as EGCG did not inhibit HhaI- or XPG-mediated incision of the DNA substrate. EGCG: (-)-epigallocatechin-3-gallate; IC_50_: half maximal inhibitory concentration.

Several recent developments have been made in identifying small molecular inhibitors of ERCC1/XPF activity. The Melton and Saxty groups have published several papers identifying several classes of molecules capable of inhibiting ERCC1/XPF activity, including catechols, 3-hydroxypyridones, N-hydroxyimides, and hydroxypyrimidones, with micromolar potency in in vitro assays [11,12,13]. In one of these studies, Compound 13 inhibited DNA repair in vitro, and led to increased γH2AX foci formation in human cells, suggesting inhibition of ERCC1/XPF in vitro [12] (Figure 1A). In another study, Compound AS5-4 disrupted ERCC1/XPF activity and enhanced cisplatin sensitivity in a melanoma cell line [13] (Figure 1A). Inhibitors of the ERCC1/XPF interaction have also been identified [13]. An in silico screen identified the compound E-X PPI2 which could disrupt the ERCC1/XPF interaction, disrupt DNA repair mediated by ERCC1/XPF, and sensitize a melanoma cell line to cisplatin [13]. In addition to these studies, we previously performed a high-throughput screen using the NCI-DTP (National Cancer Institute Developmental Therapeutics Program) diversity set and identified the compounds NSC16168 (Figure 1A) and NSC143099, which were potent and selective inhibitors of ERCC1/XPF activity both in biochemical assays as well as in lung cancer cell lines [14]. Furthermore, the compound NSC16168 was also capable of significantly enhancing tumor response to platinum-based chemotherapy in vivo [14].

In this study, we expanded upon our previous identification of the lead compound NSC143099 as an ERCC1/XPF inhibitor by performing an in silico analysis to identify molecules with structural similarities to NSC143099 that may have activity against ERCC1/XPF both in vitro and in vivo. This preliminary screen and subsequent testing identified the green tea polyphenol epigallocatechin-3-gallate (EGCG), which has approximately 90% structural similarity to the partial structure of NSC143099, as a potent, partially reversible inhibitor of ERCC1/XPF activity in vitro. Interestingly, a number of previous studies have identified EGCG as a compound capable of enhancing cisplatin sensitivity in cancer cells and in xenograft models, but the mechanism of this interaction was not established [15,16,17]. In addition, EGCG has been shown to pharmacologically inhibit 20S proteasome activity [18,19]. However, we are the first to identify the DNA endonuclease ERCC1/XPF as a pharmacological target of EGCG, helping to further explain previous observations in regard to EGCG and enhanced cisplatin sensitization. Further characterization of this compound shows it is capable of inhibiting ICL repair in vitro leading to enhanced sensitivity to cisplatin in lung cancer cell lines. Finally, we show that the inhibition of ERCC1/XPF by the EGCGprodrug, Pro-EGCG (EGCG octaacetate), could significantly enhance response to cisplatin in tumor xenografts in vivo by increasing tumor cell death and decreasing proliferation. Together, these data suggest EGCG or its prodrug may be a suitable candidate for further preclinical development as an agent capable of enhancing platinum sensitivity in tumors and overcoming drug resistance.

## 2. Materials and Methods

### 2.1. Cell Lines and Cell Culture

H1299 and H460 non-small cell lung cancer cell lines used in this study were cultured in RPMI-1640 medium (Dharmacon, Lafayette, CO, USA) supplemented with 10% Fetal Bovine Serum (Atlanta Biologicals, Flowery Branch, GA, USA) and 1% penicillin/streptomycin (Dharmacon, Lafayette). Cells were grown at 37 °C in 5% CO_2_. Cell lines were authenticated by the Biobanking and Correlative Sciences Core Facility at the Karmanos Cancer Institute.

### 2.2. In Vitro ERCC1/XPF Fluorescence Incision Assay

The in vitro ERCC1/XPF fluorescence incision assay was utilized to assess the ability of small molecules to inhibit ERCC1/XPF-mediated incision of a forked DNA substrate and was performed as previously described [14]. Purified ERCC1/XPF and XPG (Xeroderma Pigmentosum Group G) was utilized for these experiments and the purification protocol is described in [14]. The DNA substrate is described in Arora et al. [14]. Briefly, the forked DNA substrate was designed to have a 14-base dsDNA region flanked by a 12-base non-complementary ssDNA region mimicking the ssDNA:dsDNA preferred substrate for ERCC1/XPF cleavage. One strand contained a site-specific fluorescein (F) modification (5′-GCCAGCGCTCGGAT (AminoC6dT) (FLSN) TTTTTTTTTTT-3′), whereas the complementary strand contained a site-specific DABCYL quencher (Q) (5′-TTTTTTTTTTT (AminoC6dT) (Dabcyl) ATCCGAGCGCTGGC-3′) directly opposed to the fluorescein modification. An uncleaved substrate thus would not release a fluorescent signal when excited at 485 nm due to the quenching activity of the DABCYL. On the other hand, ERCC1/XPF-mediated cleavage of the forked substrate would lead to the release of the fluorescein-labeled DNA into the solution and would result in an increased fluorescent signal when measuring emission at 525 nm. The substrate was also designed with a HhaI restriction enzyme cut site in the DNA duplex, which served as a positive control for these experiments. The reactions for this experiment consisted of 10 nM DNA annealed DNA duplex, 7.5 nM ERCC1/XPF or 7.5 nM XPG in buffer (50 mM Tris-HCl pH 8.0, 2 mM MgCl_2_, 0.1 mM bovine serum albumin, 0.5 mM β-mercaptoethanol) or HhaI (20 U/mL) (New England Biolabs, Ipswich, MA, USA) and increasing concentrations of each compound prepared in DMSO. Reactions were incubated at 37 °C for 30 min after which samples were excited with a 485 nm laser and emission at 525 nm was measured using a Spectramax M5 plate reader (Molecular Devices, -San Jose, CA, USA). Data were plotted as percent fluorescence (% incision) relative to wells containing no compound.

### 2.3. Rapid Dilution Assay

Rapid dilution assays were utilized to determine the irreversible or reversible binding of the EGCG and (-)-gallocatechin gallate compounds to ERCC1/XPF. These experiments were performed as described in Liu et al. [20]. In a 10 μL reaction, the concentration of ERCC1/XPF enzyme was added at 100-fold higher than normal concentrations used in the fluorescence incision assay (7.5 nM) and then mixed with 10× the IC_90_ concentration of either EGCG, (-)-gallocatechin gallate, or diluent control (90:10, *v*/*v* DMSO:glycerol) and incubated at 37 °C for 30 min. After incubation, 2 μL of the drug-enzyme solution was diluted in 198 μL consisting of reaction buffer and the fluorescent DNA forked substrate in a 96-well plate. The reaction was monitored and the fluorescence was measured at various time points over 60 min. Data from the experiment were represented as the increase in fluorescence over time indicating the amount of activity of ERCC1/XPF on the DNA substrate.

### 2.4. Modified Alkaline Comet Assay

Modified alkaline comet assays were utilized to assess interstrand crosslink repair and were performed essentially as previously described [21,22]. H460 cells were treated with 15 M EGCG or (-)-gallocatechin gallate for two hours and then cisplatin was added to the media using the IC90 concentration for the cell line used for an additional two hours. After treatment, cells were washed with PBS and complete media was added (24 h and 48 h samples) or immediately processed for analysis at 0 h post-treatment. Prior to cell harvesting but after the experimental treatment, cells were treated with 100 μM hydrogen peroxide (H_2_O_2_) for 15 min to induce DNA double strand breaks. Following treatment with hydrogen peroxide, cells were trypsinized, pelleted, resuspended, and counted. Approximately 10,000 cells were embedded in 1% low melting point agarose and added onto slides pre-coated with a layer of 1% normal melting point agarose and allowed to solidify. A top layer of 0.5% low melting point agarose was then added and allowed to solidify. Following solidification, slides were incubated for 1 h at 4 °C in the absence of light in lysis buffer (2.5 M NaCl, 10 mM Tris, 100 mM EDTA, 1% Triton X-100, pH 10). Slides were removed from the lysis buffer and excess buffer was removed. Slides were then placed in an electrophoresis tank containing 4 °C alkaline electrophoresis buffer (300 mM NaOH, 1 mM EDTA, pH > 13), incubated for 20 min followed by electrophoresis for 30 min at 0.7 V/cm, 300 mA. Slides were removed and placed for 10 min in neutralizing buffer (0.4 M Tris-HCl, pH 7.5). Slides were stained with SYBR green (Trevigen, Gaithersburg, MD, USA) and images were taken using a Nikon epifluorescence microscope at 20 magnification. For analysis, DNA tails for at least 50 cells were measured for each slide using Komet Assay Software 5.5F (Kinetic Imaging, Liverpool, UK). Data were analyzed and quantified as in Arora et al. [14].

### 2.5. Clonogenic Survival Assays

Approximately 300–400 cells were seeded in triplicate in 60 mm dishes and allowed to attach for ~24 h. For treatments with EGCG or Pro-EGCG alone, the following day cells were titrated with the indicated concentrations of each drug for 4 h in serum-free medium followed by replacement with complete medium. For colony assays combining EGCG or Pro-EGCG with cisplatin, cells were pre-treated for 2 h and then cisplatin was added for 2 h (total treatment time with EGCG or Pro-EGCG was 4 h). After treatment, serum-free medium was replaced with complete medium. Cells were allowed to grow for approximately seven days after which plates were washed with PBS, fixed in 95% methanol, and stained in 20% ethanol containing 0.2% crystal violet dye. Colonies with >50 cells were counted using a light microscope, and percent colony survival was measured relative to the control for each group and normalized to 100%.

### 2.6. Chemicals

Cisplatin was purchased from Sigma-Aldrich (St. Louis, MO, USA) and prepared fresh prior to each experiment by making a 1 mM stock solution in PBS. (-)-epigallocatechin-3-gallate (EGCG) and (-)-gallocatechin gallate were purchased from Sigma-Aldrich and were prepared in dimethyl sulfoxide. Pro-EGCG was prepared as we previously described, and a 50 mM solution was prepared in DMSO for in vitro studies [23].

### 2.7. In Vivo Studies

For the study, 20 female athymic nude mice (five mice per group) were purchased from Taconic Biosciences (Rensselaer, NY, USA) and were maintained in accordance with protocols approved by the Wayne State University Institutional Laboratory Animal Care and Use Committee. The mice were allowed to acclimate for 1 week. Approximately 2.5 × 10^6^ H460 cells were suspended in 100 L RPMI media containing no fetal bovine serum or penicillin/streptomycin. Cells were injected subcutaneously into the right flank of each mouse. Tumor volume was measured by caliper measurements every day starting on day 3. Tumor volume was defined as (width^2^ × length/2). Weight of the mice was also measured regularly starting on the day of inoculation and through the experimental end-point. At three days post-inoculation, the mice were treated with vehicle or Pro-EGCG at 60 mg/kg by intraperitoneal IP injection. Pro-EGCG was prepared in DMSO and cremophor/ethanol (60:20:20 *v*/*v*/*v*). Once tumors reached a volume of ~100 mm^3^, cisplatin or vehicle treatment began. The mice were treated with 4 mg/kg pharmaceutical grade, sterile cisplatin three times weekly by IP injection. Drugs were prepared fresh daily. The mice were sacrificed once tumors reached ~1000 mm^3^ or at day 24, and the tumors were collected for further analysis of apoptosis and cell proliferation by immunohistochemistry.

### 2.8. Immunohistochemistry

Immunohistochemistry was performed by the Biobanking and Correlative Sciences Core at the Karmanos Cancer Institute (KCI). Subcutaneous tumors harvested from the mice were fixed in 10% formalin and were paraffin-embedded. Five μm slices were hematoxylin- and eosin-stained. Slides were stained using the Terminal nucleotidyl transferase-mediated nick end labeling assay (TUNEL) using an in situ apoptosis detection kit. Tissues were also probed for Ki67 and PCNA using standardized protocols optimized by the KCI Biobanking and Correlative Sciences Core. Images were taken at a magnification of 20. Images were quantified using ImageJ software and plotted as percent of stained area.

### 2.9. Statistical Analysis

The mouse xenograft study had five mice per treatment group, each bearing one tumor. Tumor volumes were log-transformed to meet the normality assumption, and growth curves were compared using a linear-mixed effects model with mice-specific effect as a random variable. *P* values were adjusted using Bonferroni correction. For the clonogenic survival assays, dose-response curve IC_50_ values for EGCG or Pro-EGCG in combination with cisplatin met distributional assumptions. Statistical comparisons were performed by two-sided unpaired *t*-test followed by Holm’s post-hoc analysis. Data analyses were completed using R version 3.5.0. and RStudio: Integrated Development for R (version 1.1.447, RStudio Inc.; Boston, MA, USA).

## 3. Results

### 3.1. Hits from an in Silico Screen to Identify Structural Analogues of NSC143099 with Activity Against ERCC1/XPF Activity

We previously identified the compound NSC143099 in a high-throughput screen using the NCI-DTP diversity set, as a potent, selective inhibitor of ERCC1/XPF activity both biochemically and in vitro (Figure 1C) [14]. To expand upon the initial characterization of this compound, we performed an in silico screen to identify other structurally similar compounds that may also be capable of inhibiting ERCC1/XPF activity. A search for compounds having 80% or higher structural similarity to NSC143099 was conducted for all commercially available compounds listed in the Chemical Abstract Service (CAS) database using the online SciFinder^®^ search tool (CAS, Columbus, OH, USA), whereas a locally available compound library established in the University of Toledo Center for Drug Design and Development (approximately 1000 compounds synthesized or purchased for multiple projects) was searched using ChemBioFinder Ultra (v14.0, Cambridge Soft Corp, Perkin Elmer Inc., Waltham, MA, USA). No hits were identified at this level of similarity from either source for the full NSC143099 molecule. However, a similarity search using just half of the molecule (the three-ring partial structure repeated in the upper and lower sections of the molecule) did identify six molecules available commercially or in the local compound library with a similarity of greater than 85% [24]. All six of these agents were surveyed in our frontline screening assay. From the screen, we identified three compounds with nanomolar potency against ERCC1/XPF activity in an in vitro, fluorescence-based ERCC1/XPF DNA-incision assay. The ERCC1/XPF incision assay consists of a forked DNA substrate sharing a 14-base complementary region followed by a 12-base non-complementary region (Figure 1B). The substrate contains both a fluorophore and quencher directly opposed to one another such that if a nuclease cleaves the substrate, the fluorophore will be released into solution and release a fluorescent signal when excited at 485 nm. This Y-shaped forked DNA structure represents an optimal substrate for ERCC1/XPF cleavage as shown in Arora et al. [14]. Additionally, the DNA substrate has a HhaI restriction enzyme cleavage site allowing for the use of HhaI as a positive control as well as assessing indiscriminate inhibition of compounds against an unrelated DNA endonuclease. Finally, because of the forked nature of the DNA substrate, it also serves as a prime substrate for cleavage by XPG, allowing us to evaluate off-target inhibition of a related DNA endonuclease. All three identified compounds share structural similarities to NSC143099 (Figure 1C). Myricetin, (-)-epigallocatechin-3-gallate (EGCG), and (-)-gallocatechin gallate (GCG) all inhibited ERCC1/XPF activity in vitro (IC_50_s ranging from ~40–150 nM) (Figure 1C). Myricetin is known to have multiple targets in mammalian cells, including the DNA endonuclease, Ape1, (25), so we did not further assess myricetin’s ERCC1/XPF inhibitory activity. Notably, EGCG and GCG had potent inhibitory activity against ERCC1/XPF-mediated DNA incision, while having no activity against HhaI or XPG (Figure 1D).

### 3.2. EGCG Is a Partially Reversible ERCC1/XPF Inhibitor Capable of Blocking Interstrand Crosslink Repair In Vitro

**Figure 2:**
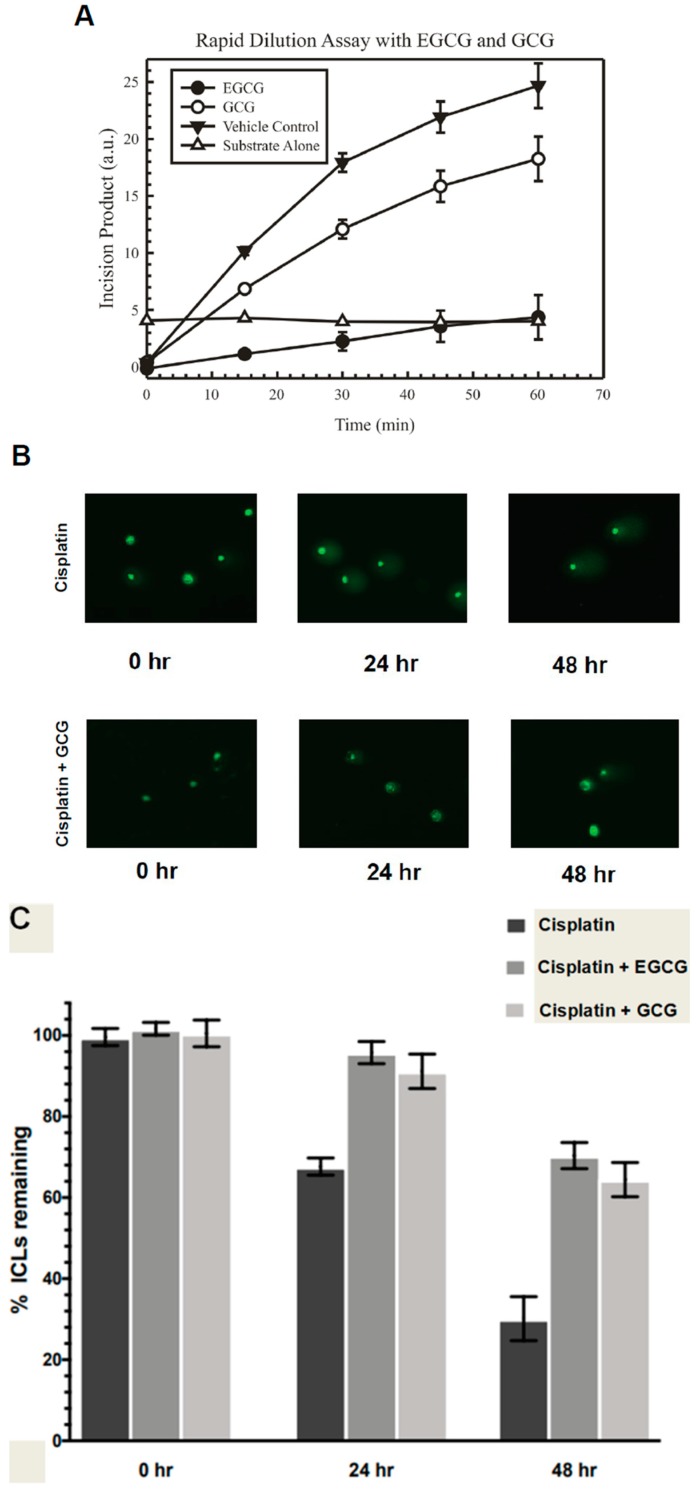
(**A**) Results from the rapid dilution assay showing GCG is a reversible inhibitor of ERCC1/XPF and EGCG is either partially reversible with slow kinetics or is irreversible. Data represented as average ± standard deviation. (**B**) Representative images of modified alkaline comet assay results for cisplatin and cisplatin + GCG in H460 cells. (**C**) Quantified data from the modified alkaline comet assay resulted in H460 cells showing inhibition of interstrand crosslink repair in cells treated with cisplatin + EGCG or cisplatin + GCG. GCG: (-)-gallocatechin gallate.

After identifying EGCG and GCG as structurally similar compounds to NSC143099 with inhibitory activity against ERCC1/XPF, we assessed the reversibility of the inhibition in vitro using a rapid dilution assay. The rapid dilution assay allowed us to assess whether the inhibition of ERCC1/XPF by EGCG or GCG was reversible in nature. The initial reaction consisted of 100ERCC1/XPF enzyme and the IC_90_ concentration of either compound determined in the ERCC1/XPF incision assay followed by a 30 min incubation at 37 °C to allow for compound binding to the enzyme. Following this initial incubation, the reaction was diluted 100-fold into a solution containing buffer and the fluorescent DNA substrate. Fluorescence was monitored over time to assess whether the enzyme recovered incision capability or if this activity was continually inhibited after dilution. As a positive control, adding ERCC1/XPF to the DNA substrate increased the fluorescent signal over time (Figure 2A). It appears that GCG is a nearly completely reversible inhibitor of ERCC1/XPF activity, as dilution of the enzyme/drug solution led to an increased fluorescent signal over time very similar to the addition of ERCC1/XPF alone to the reaction (Figure 2A). On the other hand, the rapid dilution of EGCG/enzyme solution led to a slight increase in fluorescent signal over time, suggesting that this inhibitory activity of the compound may be partially, but incompletely reversible with slow kinetics of reversibility (Figure 2A). EGCG and GCG are diastereomers, namely, they have identical structures but differ in their stereochemistries. EGCG, the most abundant polyphenol in green tea extract, has the 2S, 3S configurations with the two groups cis- to each other, whereas (-)-GCG has the 2R, 3S configuration with the two groups trans- to each other (Figure 1B). These data may indicate that the stereochemistry of the benzenetriol group at the 2-position may influence the reversibility of the inhibition of ERCC1/XPF activity by these natural compounds.

Next, we assessed the ability of EGCG and GCG to inhibit repair of cisplatin-induced interstrand crosslinks in human cancer cells via a modified alkaline comet assay. Because ERCC1/XPF endonuclease activity is critical for repair of DNA interstrand crosslinks, we chose to assess the ability of these compounds to inhibit repair of these DNA lesions. Furthermore, the modified alkaline comet assay is an established method to indirectly monitor interstrand crosslink repair over time in cells. H460 lung cancer cells were treated with 15 μM EGCG or GCG for 2 h, after which an IC_90_ concentration of cisplatin was added for an additional 2 h followed by drug removal and replacement with complete medium. Just following the experimental treatment and time-course, cells were treated with 100 μM H_2_O_2_ to induce random DNA double strand breaks, a critical component of the assay to be able to distinguish ICL containing from repaired DNA segments during denaturing electrophoresis. Immediately after initial treatment, single cell electrophoresis and staining of the DNA was performed and the level of interstrand crosslinking was determined and normalized to 100% interstrand crosslinks remaining for each treatment group (Figure 2B,C). Then, 24 h post-treatment, we observed a decrease in the amount of ICLs remaining as evident by the increased tail length over time, as observed in the comet assay images which were indicative of increased ICL repair over time (Figure 2B,C). Persistence of ICLs in this assay resulted in a maintenance of shorter tail length as the crosslink retarded the DNA mobility. This trend continued into the 48 h time point (Figure 2B,C). On the other hand, treatment with EGCG or GCG led to decreased repair of DNA ICLs over time which was observed starting at the 24 h time point and continuing into the 48 h time point (Figure 2B,C). Together these data indicate that EGCG is a potentially partially reversible inhibitor of ERCC1/XPF and GCG is a reversible inhibitor of ERCC1/XPF activity; however, both compounds are capable of inhibiting ICL DNA repair in vitro.

### 3.3. EGCG and Pro-EGCG Enhance Cisplatin Sensitivity in Lung Cancer Cell Lines In Vitro

**Figure 3:**
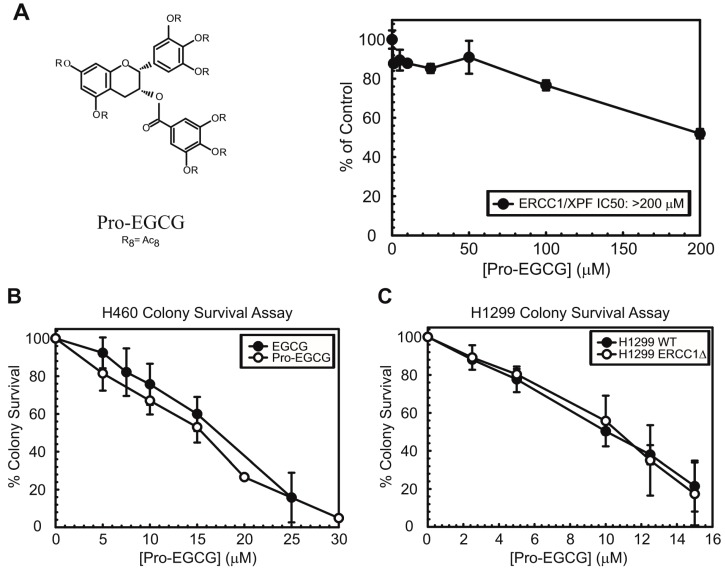

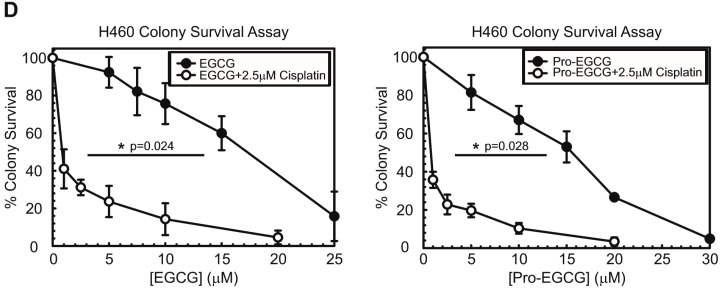
(**A**) Structure (Left) and activity (Right) of Pro-EGCG, the EGCG prodrug containing acetylated hydroxyl groups which are cleaved by esterases upon entry into the cell, in the DNA-incision assay. Data represented as average ± standard deviation. (**B**) Titration of EGCG and Pro-EGCG in H460 cells showing the both reduce clonogenicity to approximately the same extent. (**C**). Inhibition of clonogenicity by Pro-EGCG as a single agent appears to be independent of its targeting of ERCC1/XPF as shown by titration in H1299 wild-type and ERCC1 knockout cells. (**D**) H460 cells treated with increasing concentrations of EGCG (Left) or Pro-EGCG (Right) ± a single IC_50_ dose of cisplatin. All clonogenic assay data represented as average of experimental repeats ± standard deviation. Dose-response curves were compared by two-sided unpaired *t*-test followed by Holm’s post-hoc analysis.

After observing that EGCG and GCG could inhibit repair of ICLs in human cancer cells, we assessed the sensitivity of H460 lung cancer cells to EGCG in vitro either alone or in combination with cisplatin. For these studies, we also utilized a prodrug of EGCG, known as Pro-EGCG, which is the octaacetate of EGCG, which was first described by Lam et al. [23]. EGCG is known to have poor oral bioavailability and is subject to extensive rapid metabolic transformations [25,26]. Pro-EGCG differs from EGCG in having the reactive hydroxyl groups of EGCG converted by acetylation to peracetate-protecting groups (Figure 3A). These acetate groups are hydrolyzed back to the hydroxy groups upon entering cells of plasma due to esterases present within the cells or plasma thereby regenerating EGCG [27]. Thus, in the absence of esterases or in biochemical assays with purified protein, pro-EGCG has no inhibitory effect on ERCC1/XPF activity (Figure 3A) but showed inhibitory activity on clonogenic formation in H460 colony survival assays comparable to EGCG (Figure 3B). These inhibitory effects on colony formation are likely due to other targets of EGCG, including the proteasome [19]. This is supported by the observation that Pro-EGCG titration in H1299 wild-type and ERCC1 knockout cells led to similar inhibition of clonogenicity, indicating that the sensitivity of cells to EGCG and Pro-EGCG as a single agent is likely due to additional molecular targets (Figure 3C) [14]. It is important to note that a major genetic difference between H460 and H1299 cells is p53 status where H460 cells are p53 wild-type and H1299 cells are p53 null. We cannot exclude the possibility that p53 status may impact the sensitivity of cell lines or tumors to inhibition of ERCC1/XPF activity. However, the addition of a single ~IC_50_ dose of cisplatin in H460 cells enhanced sensitivity to EGCG and Pro-EGCG >10-fold, consistent with what we would expect if ERCC1/XPF were inhibited (Figure 3D).

### 3.4. Pro-EGCG Enhances Cisplatin Response In Vivo

**Figure 4:**
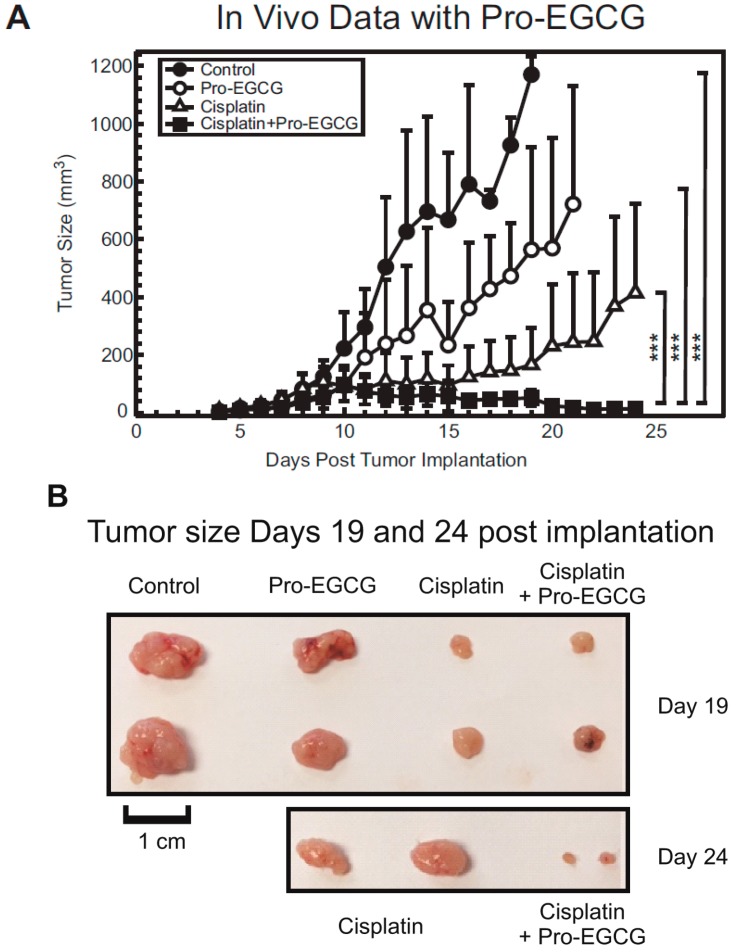
(**A**) Plot representing tumor growth of untreated, cisplatin-treated, Pro-EGCG-treated, or combination-treated mice. Data represented as tumor size (mm^3^) over time. (**B**) Images of tumors harvested from sacrificed mice at day 19 and day 24. Growth curves were compared using a linear-mixed effects model with mice-specific effect as a random variable. *P* values were adjusted using Bonferroni correction. *** *p* < 0.001.

Next, we evaluated the effect of combining Pro-EGCG with cisplatin in the treatment of H460 lung cancer xenografts. For this study, 20 female athymic nude mice (five mice per group) were inoculated subcutaneously with H460 lung cancer cells and tumors were allowed to grow. Pro-EGCG was administered daily beginning on day 3 by IP injection at 60 mg/kg. The route of administration and doses used for Pro-EGCG were similar to what we have utilized in previous studies [27]. Cisplatin treatment began once tumors reached ~100 mm^3^ and the mice were treated three times weekly with cisplatin at 4 mg/kg by IP injection. Tumor volume was measured daily by caliper measurements and plotted over time. Once tumors reached a volume of ~1000 mm^3^, the mice were sacrificed and the tumors were harvested for further analysis. Control, untreated tumors grew rapidly and all mice were sacrificed by day 19 of the experiment (Figure 4A). The addition of 60 mg/kg Pro-EGCG had some inhibitory effects on tumor growth with all mice being sacrificed by day 21. Cisplatin alone also had inhibitory effects on its own, but the combination of cisplatin and Pro-EGCG greatly enhanced these effects, with mice in the dual-treatment group having barely palpable tumors at the experimental endpoint (day 24) (Figure 4A). These inhibitory effects on tumor growth can be observed in tumors harvested from mice where at day 19 the combination-treatment group had tumors approximately the same size as the cisplatin-treated group, but by day 24, the differences in tumor size were quite dramatically different with the combination-treatment group bearing much smaller tumors than the cisplatin-treated group (Figure 4B).

### 3.5. Enhanced Cisplatin Response in Tumors Treated with Pro-EGCG Is Associated with Increased Apoptotic Markers and Decreased Cellular Proliferation

**Figure 5:**
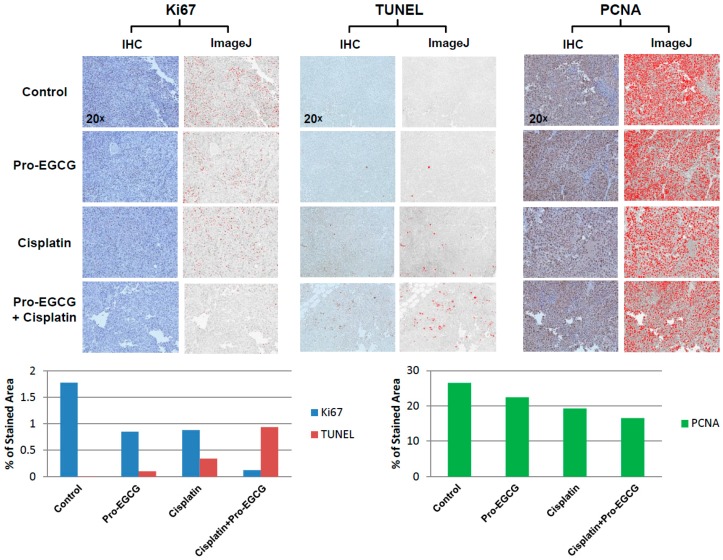
Raw images, ImageJ-processed images (**Top**), and quantification of immunohistochemical analysis of Ki67, TUNEL, and PCNA staining in tumors harvested from sacrificed mice (**Bottom**). Data showing increased TUNEL staining, decreased Ki67 staining, and decreased PCNA staining in the cisplatin+Pro-EGCG-treated tumors compared to other groups.

Tumors harvested from the mice were further processed for immunohistochemical analysis. Tissue slices were analyzed for the presence of Ki67 and PCNA to evaluate markers of cellular proliferation. In addition, TUNEL staining was performed to detect cells undergoing cell death. While the Pro-EGCG- and cisplatin-treated tumors had reduced Ki67 and PCNA staining, this effect was exacerbated in combination-treated tumors, especially in terms of Ki67 staining (Figure 5). This would be indicative of reduced tumor cell proliferation in the combination-treated group. In the context of tumor cell death, we observed the opposite effect. Pro-EGCG-treated tumors had very little increase in TUNEL staining compared to untreated, control tumors (Figure 5). This staining was increased in cisplatin-treated mice as we would expect with a cytotoxic, DNA damaging agent. However, in the combination-treated group, the increase in TUNEL staining was quite dramatic compared to the Pro-EGCG- and cisplatin-treated tumors, suggesting the combination treatment concurrently decreases the amount of cellular proliferation in the tumors and dramatically increases tumor cell death (Figure 5).

## 4. Discussion

Despite recent advances in cancer treatment such as using PD-1 and PD-L1 related therapies, platinum-based chemotherapy remains a mainstay option for a variety of tumor types either as first- or second-line therapy. Due to the prevalence of acquired or intrinsic resistance to platinum-based chemotherapy, it remains a valuable effort to identify factors that, when inhibited, can sensitize tumor cells to cisplatin. We previously have shown that ERCC1/XPF knockdown can sensitize ovarian and lung cancer cell lines to cisplatin [3]. Additionally, our group previously identified the compound NSC16168 as a potent inhibitor of ERCC1/XPF that can sensitive tumors to cisplatin [14]. The wide interest in ERCC1/XPF expression as a predictive biomarker for response to platinum-based chemotherapy also lends credence to the possibility of using inhibitors of this enzyme complex to enhance therapeutic response.

In our previous work, we performed a high-throughput screen and identified the compound NSC143099 as a potent inhibitor of ERCC1/XPF activity in vitro in both biochemical and cell-based assays [14]. In this work, we performed an in silico screen to identify other agents with structural similarity to NSC143099 that could have the potential for inhibiting ERCC1/XPF activity. From this screen, we identified three hits with nanomolar potency against ERCC1/XPF activity in a fluorescence-based DNA-incision assay: myricetin, EGCG, and GCG (Figure 1A). These compounds had substantial similarities and all shared a similar flavonoid structure. Myricetin was capable of inhibiting ERCC1/XPF activity with an IC_50_ of ~150 nM in our DNA-incision assay. However, myricetin has also been shown to inhibit a variety of other enzymes, including MEK1, PI3Kγ, and the DNA endonuclease Ape1, among others [28,29,30]. Due to the known targeting of this compound to Ape1, we did not move further with this compound in this study.

EGCG as an anti-cancer agent has been thoroughly investigated in multiple studies and in multiple cancer types, including in breast, colorectal, gastric, ovarian, and lung cancers [31]. It has been established that EGCG has multiple cellular targets including work from the Dou lab, characterizing EGCG’s inhibitory effects on the proteasome [27]. In line with these observations, treatment with EGCG or Pro-EGCG alone reduced clonogenicity in vitro in multiple lung cancer cell lines (Figure 3B). Additionally, these effects appear to be independent of any toxicity induced by inhibition of ERCC1/XPF as the sensitivity of H1299 wild-type and ERCC1 knockout cells were identical to each other (Figure 3C). Additionally, EGCG was described to have enhancing effects for cisplatin sensitivity both in vitro and in vivo. The studies assessing the cisplatin-sensitizing effects of EGCG implicated a number of different pathways and events as critical for this sensitization, including demethylation of gene promoters, increased expression of the copper transporter, CTR1, and enhancing autophagic flux [15,16,17]. In this work, we identified another target of EGCG, ERCC1/XPF, which has important implications for understanding the mechanism of the EGCG-mediated sensitization of tumors to cisplatin and for improving current cancer treatment strategies.

The EGCG compound had no activity against XPG or HhaI, suggesting its inhibition is largely specific to ERCC1/XPF (Figure 1B). Next, we showed that GCG, differing from EGCG only in the relative stereochemistry of the substitutions in the 2- and 3-positions, is a reversible inhibitor of ERCC1/XPF activity in a rapid dilution assay (Figure 2B). Conversely, EGCG is either partially reversible with slow kinetics or is an irreversible inhibitor of ERCC1/XPF activity (Figure 2A). This was similar to what we observed with another compound we identified from our original screen, NSC16168 [14]. While there were differences in the reversibility of this inhibition in biochemical assays, both EGCG and GCG could inhibit repair of cisplatin-induced interstrand crosslinks in vitro (Figure 2B,C). Not only did these compounds inhibit ERCC1/XPF activity in vitro, but this inhibition enhanced sensitivity to cisplatin in lung cancer cell lines (Figure 3). Due to the poor bioavailability and the facile metabolic transformations of EGCG, we also utilized a prodrug form of EGCG for our studies. Previous study showed that Pro-EGCG was converted intracellularly into EGCG, presumably by cellular esterases. Furthermore, Pro-EGCG was better absorbed into the cells, giving higher accumulation of EGCG by at least 2.4-fold than when the cells were treated with similar levels of EGCG [23,27]. In our DNA-incision assay, as expected, we observed that Pro-EGCG has no inhibitory effect on the purified ERCC1/XPF protein (Figure 3A). However, both EGCG and Pro-EGCG can sensitize lung cancer cells to an IC_50_ dose of cisplatin in clonogenic assays indicating these cell line models have sufficient esterase activity to convert the Pro-EGCG to active EGCG (Figure 3B).

Furthermore, after observing that EGCG could inhibit ERCC1/XPF and, along with Pro-EGCG, could sensitize lung cancer cells to cisplatin in vitro, we assessed the effects of combination treatment with cisplatin and Pro-EGCG in vivo. While Pro-EGCG and cisplatin had modest inhibitory effects on tumor growth as single agents, the combination treatment group had substantially smaller tumors at the day 24 endpoint (Figure 4A,B). The tumors in the combination treatment group were barely palpable at the experimental endpoint. Further analysis of these tumors by immunohistochemistry revealed that this sensitization was associated with the decreased presence of markers of cell growth and DNA replication and a dramatic increase in TUNEL staining indicative of cell death (Figure 5). Together these data suggest that EGCG and Pro-EGCG are potent inhibitors of ERCC1/XPF activity both in vitro and in vivo and that they are capable of sensitizing tumors to cisplatin therapy. In conclusion, we have identified the NSC143099 structural analogue, EGCG, as a potent inhibitor of ERCC1/XPF endonuclease activity capable of decreasing DNA repair in vitro and Pro-EGCG in enhancing cisplatin sensitivity in vivo. These data provide evidence that the green tea polyphenol, EGCG, and its prodrug could represent a potential structure for further pharmacological development in efforts to target the ERCC1/XPF endonuclease to enhance platinum-based chemotherapeutic response.

## 5. Conclusions

The data presented in this paper shows results from an in silico screen to identify structurally similar compounds to our lead compound NSC143099 that are capable of inhibiting ERCC1/XPF activity. The screen identified the green tea polyphenol, (-)-epigallocatechin gallate (EGCG), as a compound capable of inhibiting ERCC1/XPF activity in biochemical assays and blocking intrastrand crosslink repair in vitro. Furthermore, treatment of cells with EGCG or the prodrug form of EGCG, Pro-EGCG) was capable of sensitizing lung cancers to the chemotherapeutic agent, cisplatin. Additionally, Pro-EGCG treatment could enhance cisplatin efficacy in vivo. This increase in sensitization to cisplatin and Pro-EGCG combination treatment was correlated with decreased immunohistochemical staining of markers of cellular proliferation and increased staining for the apoptotic marker, TUNEL. Together these data suggest EGCG and its prodrug Pro-EGCG can target ERCC1/XPF activity and enhance cisplatin efficacy in vitro and in vivo.
